# Direct quantification of lipopeptide biosurfactants in biological samples via HPLC and UPLC-MS requires sample modification with an organic solvent

**DOI:** 10.1007/s00253-017-8272-y

**Published:** 2017-04-22

**Authors:** Piotr Biniarz, Marcin Łukaszewicz

**Affiliations:** Department of Biotransformation, Faculty of Biotechnology, University of Wroclaw, Joliot Curie 14a, 50-383 Wroclaw, Poland

**Keywords:** Biosurfactants, Lipopeptides, Quantification, HPLC, UPLC-MS, Extraction

## Abstract

The rapid and accurate quantification of biosurfactants in biological samples is challenging. In contrast to the orcinol method for rhamnolipids, no simple biochemical method is available for the rapid quantification of lipopeptides. Various liquid chromatography (LC) methods are promising tools for relatively fast and exact quantification of lipopeptides. Here, we report strategies for the quantification of the lipopeptides pseudofactin and surfactin in bacterial cultures using different high- (HPLC) and ultra-performance liquid chromatography (UPLC) systems. We tested three strategies for sample pretreatment prior to LC analysis. In direct analysis (DA), bacterial cultures were injected directly and analyzed via LC. As a modification, we diluted the samples with methanol and detected an increase in lipopeptide recovery in the presence of methanol. Therefore, we suggest this simple modification as a tool for increasing the accuracy of LC methods. We also tested freeze-drying followed by solvent extraction (FDSE) as an alternative for the analysis of “heavy” samples. In FDSE, the bacterial cultures were freeze-dried, and the resulting powder was extracted with different solvents. Then, the organic extracts were analyzed via LC. Here, we determined the influence of the extracting solvent on lipopeptide recovery. HPLC methods allowed us to quantify pseudofactin and surfactin with run times of 15 and 20 min per sample, respectively, whereas UPLC quantification was as fast as 4 and 5.5 min per sample, respectively. Our methods provide highly accurate measurements and high recovery levels for lipopeptides. At the same time, UPLC-MS provides the possibility to identify lipopeptides and their structural isoforms.

## Introduction

Biosurfactants (BS) are surface-active compounds of microbial origin. BS molecules consist of a hydrophobic “tail,” which is usually a fatty acid or β-hydroxyl fatty acid of 4–18 carbon atoms, and hydrophilic “head.” A wide range of molecules are considered to be BS, and classification is made primarily based on the chemical nature of the hydrophilic part of the molecule. Therefore, BS are divided into several groups: lipopeptides (LPs), glycolipids, lipoproteins, phospholipids, and polysaccharides. Over the years, BS have gained the attention of researchers around the world. BS are considered to be environmentally friendly substitutes for synthetic surfactants. Moreover, BS can act as bioemulsifiers, antibiotics, antifungals, heavy metal-binding compounds, or antitumor compounds. Therefore, BS can be utilized in different fields, such as industry, environmental protection, medicine, or farming. Potential applications of BS and LPs have been the subject of a number of reviews and research articles (Das et al. 2010; Banat et al. 2010; Janek et al. 2010, 2012; Gudiña et al. 2013; Janek et al. 2013; Duarte et al. 2014).

LPs appear to be a particularly promising class of BS because they can exhibit a variety of possible structures. The hydrophilic peptide moiety of known LPs consists of 4 to 25 amino acids, which can also form a lactone ring. The modularity of the lipopeptides’ structures results in a broad spectrum of properties and activities (Mulligan 2005; Mukherjee and Das 2010; Banat et al. 2010; Soberon-Chavez and Miller-Maier 2011; Jurado et al. 2012; Biniarz et al. 2016).

To date, LPs synthesized by different strains of *Bacillus* or *Pseudomonas* have been studied extensively, and several LP families have been mentioned in the literature: surfactins, iturins, fengycins, lychenisins, viscosins, amphisins, and several others (Raaijmakers et al. 2006; Das et al. 2010; Banat et al. 2010; Mnif and Ghribi 2015). Surfactin (SU), which is produced by a number of *Bacillus subtilis* isolates, is the best known and most extensively studied LP. SU is produced as a mixture of structural analogs that differ in the length and branching of the carbon chain, as well as in substitutions in the amino acids of the hydrophilic head. In addition, the abundance ratio of the analogs can differ among *B*. *subtilis* strains and change in response to culture conditions (Akpa et al. 2001; De Faria et al. 2011; Ben Ayed et al. 2014; Jajor et al. 2015; Mnif and Ghribi 2015). The physiochemical and biological properties of SU have been investigated extensively by many authors, revealing the antibacterial, antifungal, anticancer, heavy metal-binding, and emulsifying activities of SU (Mukherjee and Das 2010; Banat et al. 2010; Gudiña et al. 2013; Duarte et al. 2014). Research on the properties of SU is typically carried out using a mixture of SU analogs, as the separation of individual analogs can be challenging (Kowall et al. 1998; Banat et al. 2010; Tang et al. 2010). Pseudofactin (PF) is a cyclic LP that is produced by the Arctic *Pseudomonas fluorescens* strain BD5 (Janek et al. 2010). The PF molecule consists of a saturated linear fatty acid linked to a peptide head of eight amino acids. Initially, two PF analogs were characterized. PF1 (C_16_-Val_8_) and PF2 (C_16_-Leu_8_) differ in only one amino acid in the eighth position (Janek et al. 2010). *P*. *fluorescens* BD5 is also able to produce two other PF analogs, which were identified later: PF3 (C_18_-Val_8_) and PF4 (C_18_-Leu_8_) (Biniarz et al. 2015b). Of these four known analogs, PF2 is the most abundant when *P*. *fluorescens* BD5 is cultivated on minimal medium (Janek et al. 2010), but the ratio between the analogs changes in response to culture conditions (Biniarz et al. 2015b). The physiochemical and biological properties of PF2 were investigated. PF2 exhibits good emulsification activity in comparison to synthetic detergents (Janek et al. 2010), as well as antimicrobial, antiadhesive, and antibiofilm activity against several uropathogenic bacterial strains and *Candida albicans* (Janek et al. 2012; Biniarz et al. 2015a; Janek et al. 2016). Moreover, PF2 exhibits strong antitumor activity (Janek et al. 2013). There is a great need to investigate the properties of other PF analogs; therefore, methods for the exact identification and quantification of LP analogs should be established.

In recent years, extensive efforts have been made to isolate and characterize BS, as well as to investigate possible industrial applications for these molecules. However, the rapid and reliable quantification of BS remains challenging. Studies of the properties of BS, as well as the optimization of the production and utilization of BS in industry, cosmetics, drugs, etc., require fast and accurate tools for their quantification. The exact determination of the ratios between BS analogs is also of great importance.

BS have long been quantified indirectly. Several methods based on measuring changes in the surface properties of BS water solutions have been validated and utilized. These methods include surface tension measurements (Youssef et al. 2004; Joshi et al. 2013), drop-collapse assays (Youssef et al. 2004; Chen et al. 2007; Burch et al. 2010), critical micelle dilution (CMD) (Youssef et al. 2004; Satpute et al. 2008), the microplate meniscus shape assay (Chen et al. 2007), and turbidometric methods (Mukherjee et al. 2009). However, these methods can only be used as semi-quantitative techniques at best (Marchant and Banat 2014; Rudden et al. 2015; Biniarz et al. 2016). Simple colorimetric methods were also developed for the quantification of specific groups of BS and LPs. Perhaps the best known colorimetric method is the orcinol method for measuring rhamnose content in samples containing rhamnolipids. However, in this case, the results often appear to be overestimated (Marchant and Banat 2014; Rudden et al. 2015). To our knowledge, there is no colorimetric test that is specific for all LPs. Anionic BS and LPs can be detected via the blue agar plate method, but to our knowledge, this method has been utilized only as a qualitative approach (Satpute et al. 2010). Recently, a polydiacetylene (PDA) vesicle colorimetric test for the quantification of SU was developed (Zhu et al. 2014). The authors stated that PDA vesicles are a high-throughput and accurate method for the quantification of ionic surfactants (Zhu et al. 2014), but this method has been cited in a limited number of research papers to date and has not been tested for other BS.

Liquid chromatography (LC) is a powerful tool for the identification and quantification of active compounds in biological samples. Reports on the quantification of BS via reverse-phase high-performance liquid chromatography (RP-HPLC) and reverse-phase ultra-performance liquid chromatography (RP-UPLC) appeared recently (Rudden et al. 2015). LC provides researchers with highly sensitive and accurate measurements. In addition, LC enables the characterization of BS mixture components (e.g., the relative abundance of individual analogs in a mixture). LC systems can be also coupled with mass spectrometry (MS) or tandem MS (MS/MS). This modification enables the reliable identification and structural characterization of BS (Marchant and Banat 2014; Rudden et al. 2015).

Although some researchers inject LP samples directly onto HPLC/UPLC columns (Davis et al. 2001), the quantification of LPs by LC is more often preceded by laborious sample pretreatment (Rao et al. 2008). Acid precipitation (Hsieh et al. 2008; Yokota et al. 2012) or different solvent mixtures (Romero et al. 2007; Yuan et al. 2011; Yokota et al. 2012) are commonly used to extract LPs from culture supernatants. Next, the extracts are dried, dissolved in an organic solvent (usually methanol), and analyzed via LC. An organic solvent matrix provides good solubility for LPs, but this approach has several drawbacks: (1) the recovery levels of LPs from culture supernatants tend to be undefined and (2) sample pretreatment for high-throughput LC analysis should be minimized. Therefore, it is of great importance to investigate and validate quantitative methods for LPs that would minimize the sample pretreatment process. On the other hand, the direct injection of non-pretreated biological samples onto LC columns can result in adsorption issues for peptides and LPs and poor recovery levels of active substances, as well as damage to RP-HPLC columns (van den Broek et al. 2008; Yokota et al. 2012).

The aim of this study was to develop and validate fast, reliable, and simple methods for the quantification of LPs in bacterial cultures with different HPLC and UPLC-MS systems and columns, using PF and SU as standards. Three different protocols for sample pretreatment were tested. In direct analysis (DA), bacterial cultures were clarified by centrifugation and then directly injected and analyzed by LC. As a modification of DA, the clarified samples were diluted with methanol (MeOH). We showed that the recovery levels of LPs during HPLC and UPLC-MS analysis depend on the concentration of LPs in aqueous samples and the solvent mixture used to dissolve or dilute the sample. As an alternative to DA, freeze-drying followed by solvent extraction (FDSE) was tested for the analysis of “heavy” samples, as a high protein concentration can be damaging to RP-HPLC/UPLC columns (van den Broek et al. 2008). In FDSE, clarified bacterial cultures were freeze-dried, and the resulting powder was extracted with different solvents. Then, the organic extracts were analyzed via LC. The influence of solvent extraction on LP recovery was evaluated.

To our knowledge, this work is the first time that DA and FDSE have been developed and validated for the quantification of LPs via HPLC and UPLC-MS. Moreover, the influence of the LP concentration and MeOH addition to the sample on the recovery of LPs have been evaluated, highlighting the need for sample dilution with MeOH prior to LC analysis.

## Materials and methods

### Strains and culture conditions

*P*. *fluorescens* BD5 (PCM B/00115) (Janek et al. 2010) and *B*. *subtilis* Natto KB1 (PCM B/00114) isolates (both from the Laboratory of Biotransformation, University of Wroclaw culture collection) were grown on Luria-Bertani agar plates (LB; 10 g/L of tryptone, 5 g/L of yeast extract, and 10 g/L of NaCl) at 30 °C. Next, single colonies from the agar plates were used to inoculate 10 ml of liquid precultures in LB medium. The precultures were incubated overnight at 30 °C with agitation (180 rpm). After growth, the bacteria were pelleted (15 min., 10,000×*g*), washed twice with 0.9% NaCl, and resuspended in 5 ml of 0.9% NaCl. The optical density (OD) at 600 nm was measured with a Hach Oddyssey DR/2500 spectrophotometer, and suspensions were used to inoculate cultures for LP production.

### Production of lipopeptides

The PF-producing strain *P*. *fluorescens* BD5 was cultivated in King’s B medium: 20 g/L of proteose peptone (Becton Dickinson, USA), 1.5 g/L of K_2_HPO_4_ (POCH, Poland), 1.5 g/L of MgSO_4_ × 7H_2_O (POCH, Poland), and 100 mM MOPS (Bioshop, Canada), supplemented with 4% (*w*/*v*) glycerol (VWR International, USA). The SU-producing strain *B*. *subtilis* KB1 was cultivated in modified Landy’s medium: 20 g/L of glucose (POCH, Poland), 2.3 g/L of (NH_4_)_2_SO_4_ (POCH, Poland), 2 g/L of glutamic acid (POCH, Poland), 1 g/L of yeast extract (Becton Dickinson, USA), 0.5 g/L of MgSO_4_ (POCH, Poland), 0.5 g/L of KCl (POCH, Poland), 1.6 mg/L of CuSO_4_ (POCH, Poland), 1.2 mg/L of Fe_2_(SO_4_)_3_ (POCH, Poland), 0.4 mg/L of MnSO_4_ (POCH, Poland), and 100 mM (Bioshop, Canada) (Guez et al. 2008). The cultures were inoculated to achieve an OD of 0.1 and incubated for 3 days at 30 °C with agitation (180 rpm).

Three different Erlenmeyer flasks and filling volumes were used to culture the bacteria to achieve various oxygenation levels, resulting in different concentrations of LPs at the end of the incubation period: 400 ml of medium in a 1 L flask (culture A), 200 ml of medium in a 1 L flask (culture B), and 100 ml of medium in a 0.5 L baffled flask (culture C).

At the end of the incubation period, the cultures were centrifuged (15 min, 10,000×*g*), and the supernatants were used for the designated experiments.

### Preparation of lipopeptide standards

PF was produced and purified as described previously (Janek et al. 2010), with modifications. Briefly, 500 ml of BD5 cell-free culture supernatant (culture A) was extracted three times with ethyl acetate. The solvent was evaporated under a vacuum, and the crude extract was dissolved in MeOH and purified via RP-HPLC. Semi-preparative RP-HPLC consisted of a Beckman Coulter System Gold 126NMP Pump and a Knauer Variable Wavelength Monitor equipped with a Phenomenex Luna C18(2) column (100 mm × 30 mm, 10 μm) under the control of the LP-Chrom software (Lipopharm, Poland). Mobile phases of water with 0.1% (*v*/*v*) TFA (A) and acetonitrile (ACN) with 0.1% (*v*/*v*) TFA (B) were used. The absorbance at 210 nm was monitored during HPLC preparation. Two milliliters of sample were injected onto a column and eluted with a 40-min gradient (% A:B *v*/*v*): injection start (30:70), 5 min (30:70), 10 min (10:80), 20 min (20:80), 21 min (0:100), 31 min (0:100), 32 min (30:70), and 40 min (30:70). The flow rate was set at 10 ml/min. All observed PF fractions were collected together, freeze-dried, and weighed. PF was dissolved in MeOH and used as a standard stock solution (1 mg/ml). The SU standard was purchased from Sigma-Aldrich (USA), dissolved in MeOH and used as a standard stock solution (1 mg/ml).

### HPLC and UPLC-MS conditions

Three different HPLC and UPLC-MS systems were used for the quantification of LPs. System 1 (HPLC) consisted of a Beckman Gold 126 Pump and a Knauer Variable Wavelength Monitor equipped with a Macherey-Nagel C18 Isis column (50 mm × 4.6 mm, 1.8 μm) under the control of the LP-Chrom software (Lipopharm, Poland). The column was kept at room temperature during the analyses. System 2 (HPLC) consisted of a Waters e2695 pumping module with an autosampler and a 2998 PDA detector, equipped with a Waters C18 Xbridge column (50 mm × 4.6 mm, 2.5 μm). The column was kept at room temperature during the analyses. System 3 (UPLC-MS) consisted of a Waters Acquity UPLC System with a 2996 PDA detector and a Waters Xevo QTof MS System, equipped with a Waters Acquity BEH C18 column (100 mm × 2.1 mm, 1.7 μm), which was kept at 40 °C. Mobile phases of water with 0.1% (*v*/*v*) TFA (A) and ACN with 0.1% (*v*/*v*) TFA (B) were used. The absorbance at 210 nm (system 1) and the absorbance between 200 and 400 nm (system 2) were monitored during the HPLC analyses. In the case of UPLC-MS analyses, the absorbance between 200 and 400 nm was monitored simultaneously with the total ion count (TIC). MS analysis was conducted in positive mode ESI. The source temperature was set to 150 °C, and the desolvation gas temperature was 350 °C. Nitrogen was used as the desolvation gas (800 L/h) and the cone gas (20 L/h). The cone voltage was set to 10 V, and the capillary voltage was set to 3 kV. The samples were analyzed in the range of 800–1200 *m/z*.

The elution methods used for the quantification of LPs are described below. For system 1, 10 μl of sample were injected onto a column and eluted with two different gradients (% A:B *v*/*v*). A 15 min gradient was used for PF: injection start (30:70), 6 min (10:90), 8 min (0:100), 10 min (0:100), 12 min (30:70), and 15 min (30:70). The flow rate was set to 1 ml/min. For SU, a 20 min gradient was used: injection start (30:70), 2 min (20:80), 12 min (10:90), 14 min (0:100), 16 min (0:100), 18 min (30:70), and 20 min (30:70). The flow rate was set to 1.5 ml/min. For system 2, 50 μl of sample was injected onto a column and eluted with gradients similar to those used for system 1. For system 3, 5 μl of sample was injected onto a column. The elution method for PF was a 4 min gradient (% A:B) with a flow rate of 0.7 ml/min: injection start (30:70), 0.5 min (30:70), 2.5 min (20:80), 3.0 min (0:100), 3.5 min (30:70), and 4.0 min (30:70). For the analysis of SU, the flow rate was set to 0.6 ml/min with a 5.5 min gradient, as follows: injection start (50:50), 0.1 min (50:50), 1.5 min (20:80), 3.5 min (10:90), 4.5 min (0:100), 5.0 min (50:50), and 5.5 min (50:50).

### Calibration and validation of HPLC and UPLC-MS methods

PF and SU stock solutions in MeOH were used to prepare a dilution series of LPs from 0.98 to 1000 mg/L in MeOH. These samples were injected onto HPLC and UPLC columns and eluted as described above. Each sample was prepared in triplicate and analyzed at least three times. The retention times and peak areas were collected from the HPLC systems for quantification. From the UPLC-MS system, the retention times, peak areas, and TIC at a given *m*/*z* were collected. The results were used to prepare standard curves for LP quantification and to determine standard deviations (SD), relative standard deviations (RSD), limits of detection (LOD), and limits of quantification (LOQ). For the purpose of PF and SU quantification, all detected PF or SU peaks were integrated and summed. The LOD and LOQ were determined based on a visual evaluation of chromatograms, as suggested by the United States Food and Drug Administration (US FDA 1999).

### Recovery tests

Three different methods were tested for the HPLC and UPLC-MS quantification of LPs. In DA, LP-containing solutions were clarified by centrifugation (15 min, 10,000×*g*) and injected onto HPLC and UPLC-MS columns. Optionally, the samples were diluted with water or MeOH before injection. For the analysis of FDSE efficiency, 1 ml of supernatants were freeze-dried and then extracted three times with 1 ml of MeOH, ethanol (EtOH), butanol (ButOH), ACN, or ethyl acetate (EtOAc). Next, the solvents were evaporated under a vacuum, and resulting pellet was resuspended in MeOH and used for further HPLC and UPLC-MS analyses.

Recovery tests for DA were performed as follows: 50, 100, and 250 μg of PF or SU from stock solutions were added to Eppendorf test tubes and dried. Then, LB or LB:water (50:50 *v*/*v*) or LB:MeOH (50:50 *v*/*v*) mixtures were added to a final volume of 1 ml. Next, the samples were injected onto HPLC and UPLC-MS columns and analyzed as described above. Similarly, different mixtures of LB:MeOH (90:10, 80:20, 60:40, 40:60, 20:80, and 10:90 *v*/*v*) were used to dissolve 50 and 250 μg of PF or SU from stock solutions to show the effect of the MeOH concentration on LP recovery.

For the analysis of FDSE efficiency, 250 μg of PF or SU from stock solutions were added to Eppendorf test tubes and dried. Then, LB was added to a final volume of 1 ml. The samples were freeze-dried, and the resulting pellet was extracted three times with 1 ml of MeOH, EtOH, ButOH, ACN, or EtOAc by vortex-shaking for 1 min at room temperature. Next, the solvents were evaporated under a vacuum, and the resulting pellet was resuspended in MeOH and used for further HPLC and UPLC-MS analyses. A similar protocol was also used for clarified 1-ml samples of culture supernatants after the production of LPs.

### Quantification of lipopeptides in bacterial cultures

As described in the “Production of lipopeptides” section, supernatants were used for the DA and FDSE experiments. When indicated, the samples were diluted with water (50:50 *v*/*v*) or MeOH (50:50 or 10:90 *v*/*v*) before HPLC or UPLC-MS quantification. The obtained data (retention times, peak areas, and TIC) were compared to LP standard curves.

### Data analysis

The Microsoft Excel software was used to analyze the obtained data. Means, standard deviations (SD), and relative standard deviations (RSD) were calculated. For the analysis of MS data, the Waters QuanLynx software was used. Each described sample was prepared in triplicate and analyzed at least three times.

## Results

### Preparation of LP standards

**Fig. 1:**
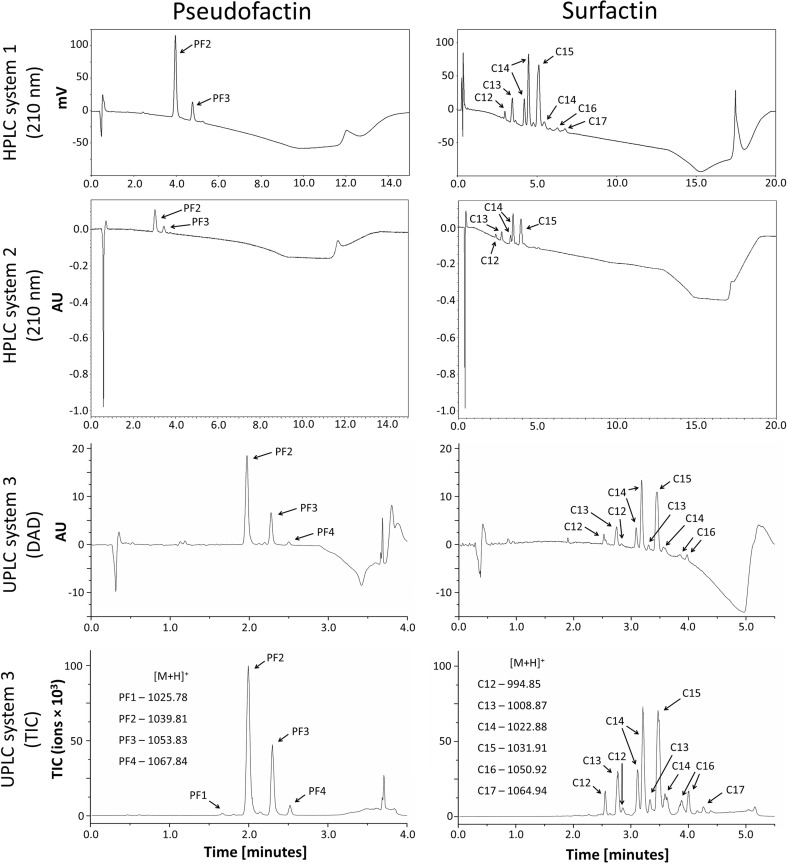
Comparison of PF and SU separation with HPLC and UPLC-MS Systems. PF and SU standards were dissolved in MeOH prior to analysis. Essential peaks are marked with *arrows* and described. The observed *m*/*z* ratios of proton adducts for LP analogs ([M + H]^+^]) are shown in the *bottom panel*

Depending on the culture conditions, PF is produced by *P*. *fluorescens* BD5 as a mixture of up to four analogs: PF1 (C_16_-Val_8_), PF2 (C_16_-Leu_8_), PF3 (C_18_-Val_8_), and PF4 (C_18_-Leu_8_). The analogs can be purified together, but the separation of each analog is also possible (Janek et al. 2010; Biniarz et al. 2015b). Similarly, SU produced by *B*. *subtilis* is a mixture of several analogs, which differ in the length of the acyl chain (from C12 to C17), as well as in amino acid substitutions (Sigma-Aldrich Surfactin-Product Information; Isa et al. 2008). The Sigma-Aldrich SU standard is sold as such a mixture (Sigma-Aldrich). The relative abundance of SU analogs was determined based on UPLC-MS TIC chromatograms (Fig. 1): (C12) 4.2%, (C13) 11.8%, (C14) 39.1%, (C15) 35.5%, (C16) 7.1%, and (C17) 2.3%. PF isolated from the culture A supernatant consisted primarily of PF2 (67.5%) and PF3 (28.0%), whereas PF1 (1.0%) and PF4 (3.4%) were observed in trace quantities (Fig. 1). The PF peaks from semi-preparative HPLC were collected together, dried, weighed, and dissolved in MeOH. These samples were then used as a PF standard for the designated experiments (Fig. 1). A mixture of PF analogs was used as a comparison to the SU mixture.

### Development of HPLC/UPLC-MS methods for LP quantification

HPLC and UPLC-MS methods for two different HPLC systems and one UPLC-MS system were developed and optimized for the separation and quantification of LPs, using PF and SU as models. Gradient elution programs allowed us to analyze PF and SU standards with a resolution sufficient for separating individual LP isoforms. Up to 4 PF isoforms and as many as 13 SU analogs were identified by the UPLC-MS system (Fig. 1). Relatively short analysis times have been achieved with conventional HPLC systems. The analysis time was only 15 min for PF and 20 min for SU, including the column wash and equilibration steps. Transferring these methods to UPLC-MS enabled 3.75-fold and 3.64-fold reductions of the analysis time for PF and SU, respectively (Fig. 1). In addition, the system 3 MS TIC allowed not only the highly sensitive detection and quantification of LPs (Table 1) but also the characterization of individual LP isoforms based on molecular mass (Fig. 1). HPLC and UPLC-MS systems showed diverse sensitivity, linearity ranges, LOD, and LOQ for LPs (Table 1). All of the tested LC systems showed broad ranges (7.81–500 mg/ml at the minimum) for PF and SU quantification when the absorbance at 210 nm was monitored. For MS TIC detection, the quantification range was narrow: between 3.91 and 125 mg/L for PF and 7.81 and 125 mg/L for SU. At the same time, the highest sensitivity (0.98 mg/L) was achieved with MS TIC detection (Table 1).

**Table 1:** Calibration data for PF and SU quantified with various LC systems

LP	LC system	Linearity range (mg/L)	*r* ^2^	LOD (mg/L)	LOQ (mg/L)
PF	System 1	3.91–1000	0.9997	1.95	3.91
	System 2	7.81–500	0.9995	1.95	3.72
	System 3 DAD	7.81–500	0.9981	3.91	7.81
	System 3 MS TIC	3.91–125	0.9958	0.98	3.91
SU	System 1	3.91–1000	0.9998	1.95	3.91
	System 2	7.81–500	0.9997	1.95	7.81
	System 3 DAD	7.81–500	0.9993	3.91	7.81
	System 3 MS TIC	7.81–125	0.9951	0.98	7.81

### Recovery of LP from standard solutions during DA

The main aim of our work was to determine the recovery levels of LPs quantified with different LC Systems. This goal was achieved by adding known amounts of LP standards to LB medium and comparing LC measurements with standard curves. Three different concentrations of LPs (50, 100, and 250 mg/ml) were used to determine whether the concentration of LPs in a sample can affect the ability to obtain reliable and quantitative results. We also investigated the influence of solvent mixtures used to dissolve LP standards. LB medium and solutions of LB:water or LB:MeOH (50:50 *v*/*v*) were used to dissolve LPs.

The LC systems showed different LP recovery levels, ranging from 0% for system 3 to 100% for systems 1 and 2 (Table 2). We detected an increase in the recovery of LPs when LB:MeOH was used to dissolve the LP standards. For example, for 50 mg/ml PF quantified with system 3 DAD, the recovery of LP was only 8.0% in LB solution; in contrast, the recovery of LP was 62.1% when LB:MeOH was used as a solvent. A similar but weaker effect was also observed for systems 1 and 2 (Table 2). Moreover, recovery was concentration-dependent, as higher recovery was observed for more concentrated LP solutions (Table 2). Systems 1 and 2 showed high and very high recovery of LPs (from 78.0 to 103.4%) when LB:MeOH (50:50) was used as a solvent, whereas only 36.3‑72.8% of the LP was detected with system 3 (Table 2). Recovery differences were also observed between PF and SU, particularly for system 3. PF in LB:MeOH, quantified with system 3, showed a 6.1‑20.0% higher recovery than SU (Table 2).

**Table 2:** Recovery of LP standards (percent) (mean ± sd, *n* = 12) dissolved in LB, LB:water, and LB:MeOH during DA. The solvent mixtures were prepared at a 50:50 (*v*/*v*) ratio. The amount of LP added to the sample was considered to be 100%. The concentration of LP was calculated by comparing the sum of the total peak areas with calibration curves for each LC system. The following HPLC/UPLC-MS configurations or monitoring protocols were used: system 1 (HPLC, absorbance at 210 nm), system 2 (HPLC, absorbance at 210 nm), system 3 DAD (UPLC-MS, absorbance at 210 nm), and system 3 MS TIC (UPLC-MS, TIC detection)

		LP concentration [mg/ml]; LP recovery [%]											
LP	Solvent	System 1			System 2			System 3 DAD			System 3 MS TIC		
		50 mg/ml (%)	100 mg/ml (%)	250 mg/ml (%)	50 mg/ml (%)	100 mg/ml (%)	250 mg/ml (%)	50 mg/ml (%)	100 mg/ml (%)	250 mg/ml (%)	50 mg/ml (%)	100 mg/ml (%)	250 mg/ml (%)
PF	LB	84.7 ± 3.6	88.3 ± 3.8	97.0 ± 4.0	67.2 ± 1.6	82.7 ± 4.3	93.8 ± 3.7	8.0 ± 0.2	8.2 ± 0.2	13.5 ± 0.7	0.0	9.3 ± 0.8	8.7 ± 0.3
	LB:water	85.8 ± 4.7	88.2 ± 2.9	95.9 ± 2.4	67.8 ± 1.3	82.0 ± 4.7	92.5 ± 1.7	13.1 ± 0.4	13.0 ± 0.2	17.2 ± 1.4	0.0	13.7 ± 0.6	12.9 ± 0.7
	LB:MeOH	95.9 ± 3.0	99.4 ± 3.6	99.9 ± 4.1	78.0 ± 1.1	103.4 ± 5.0	102.7 ± 3.3	62.1 ± 0.5	62.8 ± 1.5	66.7 ± 1.3	42.4 ± 1.4	60.1 ± 1.4	72.8 ± 1.8
SU	LB	87.8 ± 3.8	88.5 ± 3.8	95.8 ± 2.0	64.1 ± 1.8	89.0 ± 3.5	100.6 ± 2.5	6.0 ± 0.3	13.1 ± 0.3	22.8 ± 0.6	0.0	6.7 ± 0.2	16.8 ± 0.3
	LB:water	88.9 ± 4.8	89.7 ± 4.5	95.7 ± 1.7	65.6 ± 1.5	87.9 ± 3.5	99.6 ± 2.2	5.7 ± 0.3	9.5 ± 0.2	18.6 ± 0.4	0.0	4.6 ± 0.2	14.9 ± 0.4
	LB:MeOH	99.7 ± 0.5	100.9 ± 4.4	100.3 ± 1.6	80.7 ± 2.1	97.5 ± 4.8	98.4 ± 2.6	42.1 ± 1.4	49.7 ± 1.1	59.4 ± 0.5	36.3 ± 0.6	44.4 ± 0.8	54.3 ± 0.5

### Influence of MeOH concentration on LP recovery during DA

**Fig. 2:**
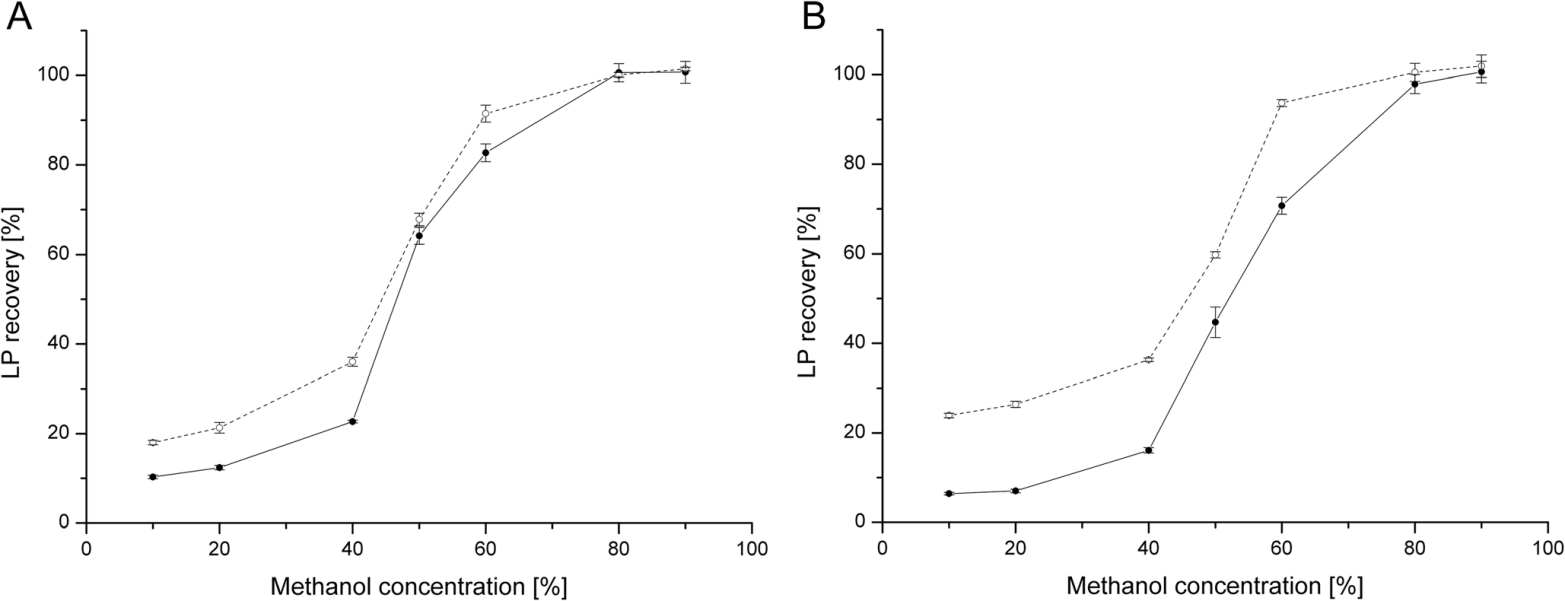
Influence of MeOH concentration (percent) on the recovery levels of PF (**a**) and SU (**b**) quantified with system 3 (UPLC-MS). Two concentrations of LPs were used: 50 mg/ml (*closed circles*, *solid lines*) and 250 mg/ml (*open circles*, *dashed lines*). LPs were quantified with DAD. Quantification with MS TIC showed similar patterns (data not shown)

UPLC-MS (system 3) showed the lowest recovery levels for LP (even when 50:50 LB:MeOH was used as a solvent) (Table 2). Therefore, we tested solvent mixtures of LB:MeOH with MeOH concentrations from 10 to 90% to investigate the influence of the MeOH concentration on the recovery levels of PF and SU quantified with system 3. We showed that the recovery of LP increases with the MeOH concentration, reaching approximately 100% when >80% MeOH was used to dissolve a sample (Fig. 2). Interestingly, for solutions with low concentration of LPs (50 mg/ml) in up to 60% MeOH, the recovery of PF was 3.8‑19.4% higher than observed for SU. The opposite effect was observed for the higher concentrations analyzed (250 mg/ml), but only for solutions containing up to 20% MeOH. Here, SU recovery was 5.1‑5.9% higher than observed for PF (Fig. 2).

### DA of LPs in bacterial cultures

PF and SU were produced by *P*. *fluorescens* BD5 and *B*. *subtilis* Natto KB1, respectively. Three oxygenation levels were used to culture the bacteria, resulting in various final LP concentrations in the cultures (Table 3). This approach allowed us to avoid introducing differences in the composition of the culture media between samples. Next, the samples were clarified, and LPs were quantified with the LC systems. To determine the influence of the MeOH concentration on LP quantification directly in the bacterial cultures, we quantified LPs in samples diluted two times with water (2× water) and two or ten times with MeOH (2× MeOH and 10× MeOH). The results were compared to undissolved (undiluted with MeOH) samples (1× sample), which were set as 100%.

**Table 3:** Relative recovery of LPs (percent) (mean ± sd, *n* = 9) from culture broths diluted with water (2× water) or MeOH (2× MeOH and 10× MeOH) in comparison to LPs quantified in undissolved samples (1× sample, 100%). The concentration of LPs was calculated by comparing the sum of the total peak areas with calibration curves for each LC system. The following HPLC/UPLC-MS configurations or monitoring protocols were used: system 1 (HPLC, absorbance at 210 nm), system 2 (HPLC, absorbance at 210 nm), system 3 DAD (UPLC-MS, absorbance at 210 nm), and system 3 MS TIC (UPLC-MS, TIC detection)

LP	Culture	LP concentration (mg/l)^a^	Sample dilution: undissolved sample (1×), diluted with water (2×), or diluted with MeOH (2× and 10×); LP recovery (%)												
			1× sample (%)	System 1			System 2			System 3 DAD			System 3 MS TIC		
				2× water (%)	2× MeOH (%)	10× MeOH (%)	2× water (%)	2× MeOH (%)	10× MeOH (%)	2× water (%)	2× MeOH (%)	10× MeOH (%)	2× water (%)	2× MeOH (%)	10× MeOH (%)
PF	A	72.5 ± 3.1	100.0	97.4 ± 3.9	108.9 ± 4.7	115.9 ± 2.0	86.8 ± 0.8	101.0 ± 1.4%	Nd	40.1 ± 0.9	140.3 ± 3.8	324.9 ± 5.8	52.7 ± 2.0	203.8 ± 4.7	372.6 ± 12.7
	B	97.2 ± 4.9	100.0	96.2 ± 3.8	110.5 ± 5.5	117.3 ± 1.2	83.4 ± 2.4	98.5 ± 1.5%	Nd	66.0 ± 1.9	234.7 ± 5.9	609.1 ± 6.7	66.7 ± 2.4	244.0 ± 4.1	492.8 ± 4.5
	C	504.3 ± 3.9	100.0	98.3 ± 1.2	102.1 ± 0.8	106.8 ± 0.5	85.7 ± 1.6	98.5 ± 1.1%	Nd	30.3 ± 1.2	135.4 ± 0.7	542.5 ± 6.3	66.4 ± 2.5	214.0 ± 1.8	564.7 ± 2.3
SU	A	219.1 ± 7.5	100.0	96.6 ± 1.4	110.7 ± 3.8	119.0 ± 1.0	96.8 ± 2.4	109.0 ± 2.7%	Nd	79.4 ± 0.7	162.5 ± 4.2	315.0 ± 3.7	56.4 ± 0.4	174.3 ± 1.4	318.6 ± 1.9
	B	455.7 ± 9.0	100.0	97.6 ± 2.5	104.4 ± 2.1	107.2 ± 1.0	74.8 ± 2.7	109.5 ± 2.4%	Nd	77.2 ± 1.6	163.7 ± 4.1	422.5 ± 3.4	53.4 ± 0.5	189.0 ± 0.4	490.2 ± 1.3
	C	1154.0 ± 49.2	100.0	97.1 ± 4.1	109.2 ± 4.7	116.1 ± 0.6	97.3 ± 0.7	108.3 ± 0.8%	Nd	87.1 ± 0.3	268.8 ± 2.9	371.9 ± 2.7	65.9 ± 0.1	128.1 ± 2.9	353.4 ± 0.9

*Nd* no data

^a^LPs were quantified with the system 1 HPLC for samples diluted 2× with MeOH

We detected an increase in LP recovery levels (in comparison to undissolved samples) when the samples were diluted two times or ten times with MeOH. For system 1, a 2× dilution with MeOH resulted in a 2.1‑10.7% higher recovery of LPs, while a 10× dilution with MeOH increased the recovery of LPs from 6.8 to 19.0% in comparison to undissolved samples (Table 3). The influence of MeOH addition was intense for system 3. We observed approximately 1.3 to 2.7 times higher concentrations of LPs for samples diluted 2× with MeOH in comparison to undissolved samples when using system 3 DAD. Even more dramatically, a 3.5- to 6.1-fold higher concentration of LPs (in comparison to undissolved samples) was observed when the samples were diluted 10× with MeOH prior to system 3 DAD analysis (Table 3).

### Efficiency of FDSE for sample pretreatment during quantitative analysis of LPs

We also tested the efficiency of freeze-drying followed by solvent extraction (FDSE) for the quantitative analysis of LPs in biological samples. FDSE was developed as a rapid LP extraction protocol that can be used prior to quantitative LC analysis of LPs. The sample pretreatment process can potentially minimize RP-LC column damage (e.g., by proteins) and as a result, extend the column lifespan.

Similar to DA, LP standards (250 mg/ml) from stock solutions were added to LB medium. Then, the samples were freeze-dried, and the resulting pellet was extracted three times with equal volumes of MeOH, EtOH, ButOH, ACN, or EtOAc. Next, the solvents were evaporated, the pellet was resuspended in MeOH, and the samples were analyzed using system 3 DAD.

**Fig. 3:**
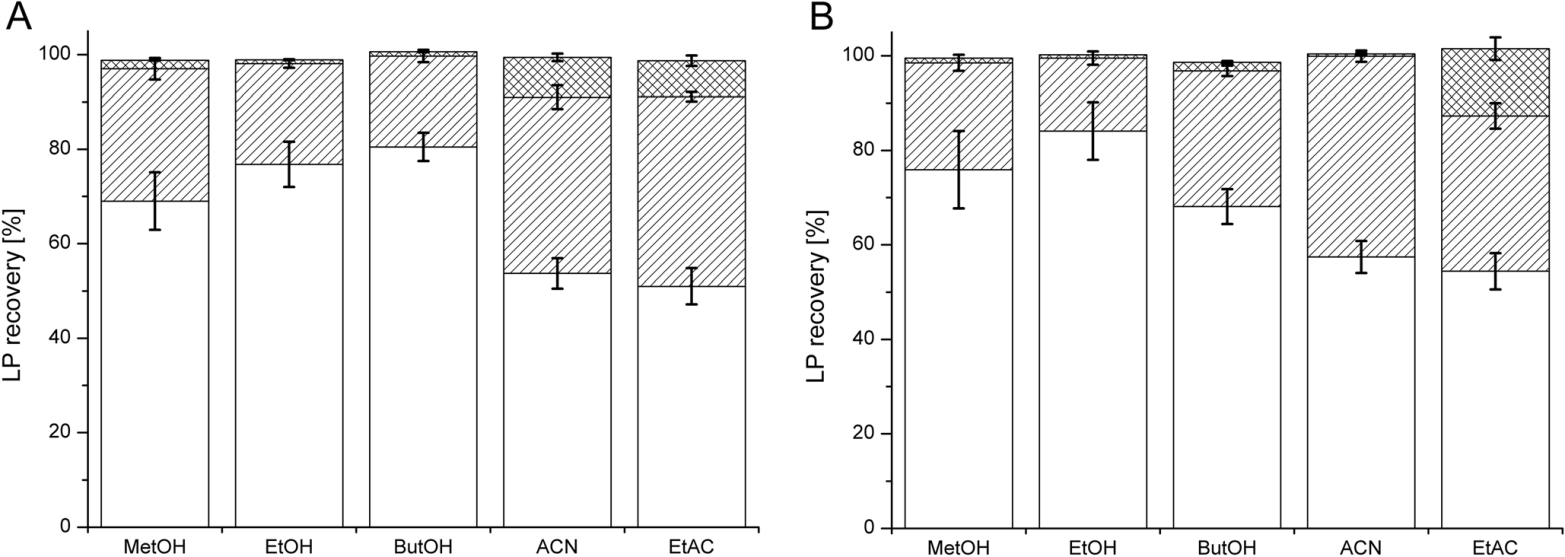
Recovery of PF (**a**) and SU (**b**) standards (250 mg/ml) from LB medium after FDSE. The extraction stages are shown as different patterns: first extraction (*white bars*), second extraction (*hatched bars*), and third extraction (*crossed bars*). Five solvents were used to extract LPs from lyophilized samples: methanol (MeOH), ethanol (EtOH), butanol (ButOH), acetonitrile (ACN), and ethyl acetate (EtAC). LPs were quantified with system 3 DAD

**Fig. 4:**
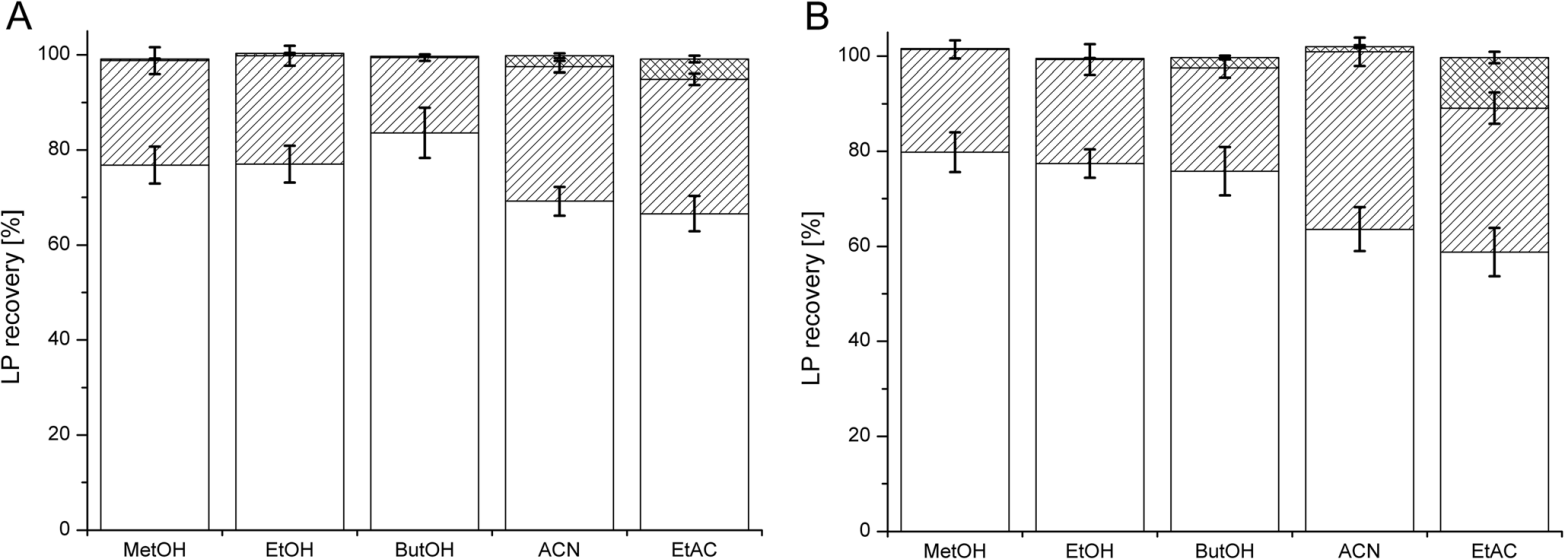
Recovery of PF (**a**) and SU (**b**) from bacterial cultures (490.0 ± 8.5 mg/L PF and 1124.6 ± 15.3 mg/L SU) after FDSE. The extraction stages are shown as different patterns: first extraction (*white bars*), second extraction (*hatched bars*), and third extraction (*crossed bars*). Five solvents were used to extract LPs from lyophilized culture samples: methanol (MeOH), ethanol (EtOH), butanol (ButOH), acetonitrile (ACN), and ethyl acetate (EtAC). LPs were quantified with system 3

The FDSE efficiency was comparable for PF extracted with MeOH, EtOH, and ButOH, reaching 69.0‑80.5% after a one-step extraction and 97.0‑99.7% after a two-step extraction. The third extraction did not influence the final extraction efficiency (Fig. 3a). ACN and EtAC were less effective after one-step extraction, but approximately 90% of the PF was extracted after the double extraction process (Fig. 3a). The recovery levels of SU extracted by MeOH and EtOH were 75.9 and 84.1%, respectively, after a one-step extraction. Nearly 100% of the SU was recovered after double extraction (Fig. 3b). EtAC was the less effective solvent for SU extraction—only 51.0, 40.1, and 7.6% of the SU was recovered after extraction (Fig. 3b). We also tested FDSE for low concentrations of LPs (50 mg/ml) and detected no significant influence of the LP concentration on the extraction efficiency by FDSE (data not shown). A similar protocol was applied for clarified bacterial cultures. Cultures of *P*. *fluorescens* BD5 and *B*. *subtilis* KB1 were analyzed. LPs in the cultures were initially quantified via system 3 (DAD) after diluting the samples 10× with MeOH (qf. Fig. 2 and Table 3). The measured concentrations were 490.0 ± 8.5 mg/ml for PF and 1124.6 ± 15.3 mg/L for SU. The FDSE efficiency values for PF and SU extracted from bacterial cultures were comparable to the FDSE efficiency values for samples containing LP standards (qf. Figs. 3 and 4). Approximately 100% of the LPs were recovered from samples double-extracted with MeOH, EtOH, ButOH, and ACN. EtAC was less effective and extracted 94.5 and 89.1% of the PF and SU, respectively, after a two-step extraction.

## Discussion

The complexity of biological matrices makes the quantification of active substances a challenging task (van den Broek et al. 2008). Over the years, BS have been quantified indirectly using various techniques, such as surface tension measurements or meniscus shape analysis, as examples. Due to the complexity of biological matrices, the results obtained with these indirect techniques are believed to be only semi-quantitative at best (Youssef et al. 2004; Burch et al. 2010; Marchant and Banat 2014; Rudden et al. 2015; Biniarz et al. 2016). Direct methods for the quantification of BS have been developed as an alternative. Perhaps the best known of these methods is the orcinol method, which has been proposed for the quantification of rhamnolipids. However, that method also suffers from crosstalk with medium components (e.g., glucose), which may lead to the overestimation of BS yields in culture medium (Marchant and Banat 2014; Rudden et al. 2015). Simple colorimetric methods were recently proposed for the detection and quantification of SU, but these methods have been cited in a limited number of research works to date (Zhu et al. 2014). Therefore, the reliable quantification of BS, particularly LPs, remains a challenging task.

LC is a powerful tool for the quantification and identification of various substances in biological samples. Recently, several reports have shown the possibility of using RP-HPLC and RP-UPLC methods for the quantification and characterization of BS with high accuracy, sensitivity, and repeatability (Hsieh et al. 2008; Rao et al. 2013; Rudden et al. 2015). LC quantification of BS is typically preceded by sample pretreatment, primarily acid precipitation or solvent extraction. Next, the extracts are dried, dissolved in organic solvent (usually MeOH), and analyzed via LC (Hsieh et al. 2008; Yokota et al. 2012; Marchant and Banat 2014; Zhu et al. 2014). These protocols are time-consuming and are therefore not applicable for high-throughput analysis. In addition, the recovery levels of BS usually remain unknown (Hsieh et al. 2008; Ke et al. 2015; Geissler et al. 2016).

Recently, LC and LC-MS methods for the quantification of rhamnolipids have been proposed and validated (Rudden et al. 2015). There are also a few examples of different protocols for sample pretreatment prior to LC quantification of LPs. In addition, method validation has been carried out in some cases. Yokota et al. quantified iturin A with HPLC and compared the recovery levels of this LP extracted from *Bacillus* culture supernatants using three methods: acid precipitation and MeOH extraction (APME), ButOH extraction and MeOH substitution (BEMS), and DA. Those authors showed poor recovery levels of iturin A for DA and APME (0.5 and 14.1%, respectively) and high efficiency for BEMS (99.6%) (Yokota et al. 2012). In contrast, Mubarak et al. showed that the recovery levels of SU quantified directly (DA) by HPLC were 94.1‑102.4%, but no information concerning the HPLC system used or the protocol used to prepare the samples for the recovery tests were included (Mubarak et al. 2015). Bie et al. used traditional acid precipitation to pellet raw antimicrobial substances produced by *Bacillus* sp. fmbJ and then tested four solvents (MeOH, EtOH, propanol, and ButOH), as well as various pH levels and time periods, for the optimal extraction of active substance(s). Those authors found MeOH and EtOH to be efficient solvents. In addition, the pH and extraction time also affected the extraction efficiency (Bie et al. 2005). Liquid-liquid extraction (LLE) is widely used for the semi-preparative purification of LPs (Janek et al. 2010; Smyth et al. 2014), but only in a few research works LLE has been applied for analytical scale extraction. The reasons for this limitation are primarily poor recovery and the complexity of small-scale LLE (Smyth et al. 2010; Burch et al. 2010). An aqueous two-phase system (ATPS) was previously tested as an alternative to liquid-liquid extraction (LLE) to overcome the poor recovery of LLE (Yuan et al. 2011). Iturin A was quantified with good resolution, a relatively short analysis time, and high accuracy and recovery (up to 97%), but the simplicity and feasibility of using the proposed method in high-throughput quantification is, in our opinion, arguable (Yuan et al. 2011). Solid-phase extraction (SPE) has been also implemented for the quantification of LPs in bacterial cultures. Here, C_8_ or C_18_ SPE columns are typically used. Clarified culture supernatants are applied to the SPE column, and LPs are then eluted with MeOH. Then, the methanolic extracts are analyzed via LC (Gancel et al. 2009; Coutte et al. 2010).

In our work, we developed and validated a simple method for sample pretreatment (FDSE). Several dozen samples can be lyophilized and extracted in only a few hours. In addition, except the lyophilizer, no specialized equipment or consumables (such as an SPE chamber or SPE columns) are needed. At the same time, FDSE provides high recovery levels for LPs (more than 97% for samples twofold extracted with MeOH, EtOH, ButOH, and ACN) and good repeatability. Therefore, this method can be used for the extraction of LPs from cultivation medium prior to LC or LC-MS identification and LP quantification.

Sample pretreatment complicates and increases the cost of LP quantification and therefore should be minimized in high-throughput optimization of LP production or LP analysis in the food industry or healthcare products. The direct injection of microbial cultures onto LC columns for the quantification of LPs appears to be the solution to this problem, but this approach has been mentioned only in a few research works (Lin et al. 2007; Isa et al. 2007; Rao et al. 2008; Yokota et al. 2012; Yi et al. 2016). Thus, DA of LPs should be validated, especially because recovery problems have been reported previously (Yokota et al. 2012). Simple dilution of the sample with an organic solvent (e.g., MeOH or EtOH) could be a solution. Sample dilution with MeOH followed by DA via HPLC was tested for iturin A quantification (Rao et al. 2008). Culture samples were clarified by centrifugation and filtration. Then, the cell-free supernatant was passed through 1000 and 10 kDa ultrafiltration membranes, and the concentrated fraction was diluted ten times with methanol and analyzed via HPLC (Rao et al. 2008). In another report, those authors also diluted the samples ten times with a different solvent system (acetonitrile: 10 mM ammonium acetate, 40:60 *v*/*v*) and omitted the ultrafiltration steps (Lin et al. 2007). HPLC methods showed good resolution for iturin A isomers. In addition, SU was directly quantified via HPLC after diluting the samples with MeOH (Yi et al. 2016). Unfortunately, no recovery tests or method validation were performed by the authors (Lin et al. 2007; Rao et al. 2008; Yi et al. 2016).

We observed that the accuracy of direct LP quantification in biological samples via LC depends on the composition of the sample injected, especially the LP concentration and the solvent mixture used to dissolve a sample (MeOH concentration in a sample). This effect can be probably explained by the loss of LPs caused by their adsorption on surfaces of the LC-MS system, as well as consumables used for sample pretreatment (e.g., pipette tips or Eppendorf tubes) (van den Broek et al. 2008). Similar effects were previously observed for bioactive peptides in various complex matrices (e.g., in human serum). The adsorption of peptides on surfaces (e.g., glass and plastic vials, pipette tips, and inner parts of LC-MS systems) is a well-known phenomenon. It was also confirmed that several factors can influence the adsorption of peptides on surfaces, primarily solvent properties (pH, ionic strength, etc.), the concentration and physiochemical properties of the peptide, temperature, and the nature of interphase (e.g., container material) (van den Broek et al. 2008). The same effects can probably also be observed for LPs, considering the similar chemical structures and physiochemical properties of various bioactive peptides and LPs. This phenomenon should be investigated in the future.

As pointed out by Rudden et al., there is a great need for the development of fast, accurate, and reliable analytical methods for the quantification of BS. In addition, such methods should be standardized between laboratories to make the BS yields reported in research works more comparable (Rudden et al. 2015). Previously, UPLC-MS/MS was developed and properly validated for the quantification of rhamnolipids (Rudden et al. 2015). To our knowledge, no LC method has been properly developed and validated for the quantification and characterization of LPs. Thus, the main objective our work was to develop such a method. Recently, high-performance and high-accuracy thin layer chromatography (HPTLC) was evaluated and validated for the simultaneous quantification of SU, iturin A, and fengycin directly from *B*. *subtilis* cultures (Geissler et al. 2016); however, in our opinion, the LC device is more ubiquitous in analytical laboratories than HPTLC. Therefore, the development of LC methods for the quantification of LPs is of great importance.

Sample analysis with HPLC can be time-consuming. This limitation also concerns the quantification of LPs. In several research papers, HPLC analysis of lipopeptides varied from 20 to up to 100 min per sample (Lin et al. 2007; Isa et al. 2007; De Bruijn et al. 2008; Yokota et al. 2012; Willenbacher et al. 2014; Zhu et al. 2014). In contrast, up to 20 samples containing SU, iturin A, and fengycin can be quantified simultaneously with HPTLC in 80 min (Geissler et al. 2016). Our HPLC methods allow us to quantify LPs in a relatively short time (15 min for PF and 20 min for SU). Transferring these methods to UPLC-MS reduced the analysis time to 4 min for PF and 5.5 min for SU. These times make our UPLC-MS methods for the quantification of LPs high-throughput, allowing the analysis of a large number of samples in relatively short time, with high accuracy and precision. Simultaneously, the use of the LC-MS system allows not only the separation of individual LPs’ structural analogs but also the precise identification of these compounds.

In our work, we also identified an issue with poor recovery of LPs quantified directly in culture samples. This observation can probably be explained by the adsorption of LPs on surfaces (e.g., plastic consumables or LC system parts). Moreover, we proposed and evaluated a simple solution for this issue, which is the modification of a sample with MeOH prior to LC analysis. Our results indicate that the MeOH concentration in the sample should reach 80% or more to completely avoid adsorption issues. Therefore, we suggest researchers working on LPs to validate their LC quantification methods, especially to investigate recovery of LPs (as shown in our work). Application of our simple method with adding MeOH to clarified culture supernatants prior quantification should be also checked for other classes of LPs. We hope that our work will be a starting point for the development of standardized and properly validated high-throughput LC methods for the quantification of LPs.

In summary, we showed:

- Novel, accurate HPLC and UPLC-MS methods for the direct quantification of lipopeptides in culture supernatants have been developed and validated, using pseudofactin and surfactin as model molecules.
- Pseudofactin and surfactin can be quantified via HPLC in 15 and 20 min per sample, while UPLC-MS reduced these times to 4 and 5.5 min per sample, respectively.
- A high accuracy of direct lipopeptide quantification via LC can be achieved by diluting the samples with methanol.
- Lipopeptides’ structural analogs can be separated and identified using a MS detector.
- Culture supernatants can be freeze-dried, and lipopeptides can be extracted from the resulting pellet with organic solvents (methanol, ethanol, butanol, or acetonitrile).

## References

[CR1] Akpa E, Jacques P, Wathelet B, Paquot M, Fuchs R, Budzikiewicz H, Thonart P, Conditions C (2001). Influence of culture conditions on lipopeptide production by *Bacillus subtilis*. Appl Biochem Biotechnol.

[CR2] Banat IM, Franzetti A, Gandolfi I, Bestetti G, Martinotti MG, Fracchia L, Smyth TJ, Marchant R (2010). Microbial biosurfactants production, applications and future potential. Appl Microbiol Biotechnol.

[CR3] Ben Ayed H, Hmidet N, Béchet M, Chollet M, Chataigné G, Leclère V, Jacques P, Nasri M (2014). Identification and biochemical characteristics of lipopeptides from *Bacillus mojavensis* A21. Process Biochem.

[CR4] Bie X, Lu Z, Lu F, Zeng X (2005). Screening the main factors affecting extraction of the antimicrobial substance from *Bacillus* sp. fmbJ using the Plackett-Burman method. World J Microbiol Biotechnol.

[CR5] Biniarz P, Baranowska G, Feder-Kubis J, Krasowska A (2015). The lipopeptides pseudofactin II and surfactin effectively decrease *Candida albicans* adhesion and hydrophobicity. Antonie Van Leeuwenhoek.

[CR6] Biniarz P, Krasowska A, Łukaszewicz M (2015). Ratio of isomers of pseudofactin, a lipopeptide produced by *Pseudomonas fluorescens* BD5, changes in response to different carbon and nitrogen sources. J Biotechnol.

[CR7] Biniarz P, Łukaszewicz M, Janek T (2016). Screening concepts, characterization and structural analysis of microbial-derived bioactive lipopeptides: a review. Crit Rev Biotechnol.

[CR8] van den Broek I, Sparidans RW, Schellens JHM, Beijnen JH (2008). Quantitative bioanalysis of peptides by liquid chromatography coupled to (tandem) mass spectrometry. J Chromatogr B Anal Technol Biomed Life Sci.

[CR9] Burch AY, Shimada BK, Browne PJ, Lindow SE (2010). Novel high-throughput detection method to assess bacterial surfactant production. Appl Environ Microbiol.

[CR10] Chen CY, Baker SC, Darton RC (2007). The application of a high throughput analysis method for the screening of potential biosurfactants from natural sources. J Microbiol Methods.

[CR11] Coutte F, Lecouturier D, Yahia SA, Leclère V, Béchet M, Jacques P, Dhulster P (2010). Production of surfactin and fengycin by *Bacillus subtilis* in a bubbleless membrane bioreactor. Appl Microbiol Biotechnol.

[CR12] Das P, Mukherjee S, Sivapathasekaran C, Sen R (2010). Microbial surfactants of marine origin: potentials and prospects. Adv Exp Med Biol.

[CR13] Davis DA, Lynch HC, Varley J (2001). The application of foaming for the recovery of surfactin from *B. subtilis* ATCC 21332 cultures. Enzym Microb Technol.

[CR14] De Bruijn I, de Kock MJD, De Waard P, van Beek T a, Raaijmakers JM (2008). Massetolide a biosynthesis in *Pseudomonas fluorescens*. J Bacteriol.

[CR15] De Faria AF, Teodoro-Martinez DS, De Oliveira Barbosa GN, Gontijo Vaz B, Serrano Silva Í, Garcia JS, Tótola MR, Eberlin MN, Grossman M, Alves OL, Regina Durrant L (2011). Production and structural characterization of surfactin (C14/Leu7) produced by *Bacillus subtilis* isolate LSFM-05 grown on raw glycerol from the biodiesel industry. Process Biochem.

[CR16] Duarte C, Gudiña EJ, Lima CF, Rodrigues LR (2014). Effects of biosurfactants on the viability and proliferation of human breast cancer cells. AMB Express.

[CR17] Gancel F, Montastruc L, Liu T, Zhao L, Nikov I (2009). Lipopeptide overproduction by cell immobilization on iron-enriched light polymer particles. Process Biochem.

[CR18] Geissler M, Oellig C, Moss K, Schwack W, Henkel M, Hausmann R (2016). High-performance thin-layer chromatography (HPTLC) for the simultaneous quantification of the cyclic lipopeptides Surfactin, Iturin a and Fengycin in culture samples of *Bacillus* species. J Chromatogr B.

[CR19] Gudiña EJ, Rangarajan V, Sen R, Rodrigues LR (2013). Potential therapeutic applications of biosurfactants. Trends Pharmacol Sci.

[CR20] Guez JS, Müller CH, Danze PM, Büchs J, Jacques P (2008). Respiration activity monitoring system (RAMOS), an efficient tool to study the influence of the oxygen transfer rate on the synthesis of lipopeptide by *Bacillus subtilis* ATCC6633. J Biotechnol.

[CR21] Hsieh F-CC, Lin T-CC, Meng M, Kao S-SS (2008). Comparing methods for identifying *Bacillus* strains capable of producing the antifungal lipopeptide iturin a. Curr Microbiol.

[CR22] Isa M, Coraglia D, Frazier R, Jauregi P (2007). Recovery and purification of surfactin from fermentation broth by a two-step ultrafiltration process. J Memb Sci.

[CR23] Isa MHM, Frazier R a, Jauregi P (2008). A further study of the recovery and purification of surfactin from fermentation broth by membrane filtration. Sep Purif Technol.

[CR24] Jajor P, Piłakowska-Pietras D, Krasowska A, Łukaszewicz M (2015) Surfactin analogues produced by *Bacillus subtilis* strains grown on rapeseed cake. J Mol Struct:1–6. doi:10.1016/j.molstruc.2016.02.014

[CR25] Janek T, Łukaszewicz M, Rezanka T, Krasowska A (2010). Isolation and characterization of two new lipopeptide biosurfactants produced by *Pseudomonas fluorescens* BD5 isolated from water from the Arctic archipelago of Svalbard. Bioresour Technol.

[CR26] Janek T, Łukaszewicz M, Krasowska A (2012). Antiadhesive activity of the biosurfactant pseudofactin II secreted by the Arctic bacterium *Pseudomonas fluorescens* BD5. BMC Microbiol.

[CR27] Janek T, Krasowska A, Radwańska A, Łukaszewicz M (2013). Lipopeptide biosurfactant pseudofactin II induced apoptosis of melanoma a 375 cells by cpecific interaction with the plasma membrane. PLoS One.

[CR28] Janek T, Rodrigues LR, Gudiña EJ, Czyżnikowska Ż (2016). Structure and mode of action of cyclic lipopeptide pseudofactin II with divalent metal ions. Colloids Surfaces B Biointerfaces.

[CR29] Joshi S, Suthar H, Yadav A, Hingurao K, Nerurkar A (2013) Occurrence of biosurfactant producing *Bacillus* spp. in diverse habitats 10.5402/2013/652340 PMC4403617 25969778

[CR30] Jurado E, Altmajer D, Gudi EJ, Teixeira J a, Rodrigues LR, Vaz DA, Gudiña EJ, Alameda EJ, Teixeira J a, Rodrigues LR (2012). Performance of a biosurfactant produced by a *Bacillus subtilis* strain isolated from crude oil samples as compared to commercial chemical surfactants. Colloids Surfaces B Biointerfaces.

[CR31] Ke WJ, Hsueh YH, Cheng YC, Wu CC, Liu ST (2015). Water surface tension modulates the swarming mechanics of *Bacillus subtilis*. Front Microbiol.

[CR32] Kowall M, Vater J, Kluge B, Stein T, Franke P, Ziessow D (1998). Separation and characterization of surfactin isoforms produced by *Bacillus subtili*s OKB 105. J Colloid Interface Sci.

[CR33] Lin H, Koteswara Y, Wu W, Tzeng Y (2007). Ferrous ion enhanced lipopeptide antibiotic iturin a production from *Bacillus amyloliquefaciens* B128. Int J Appl Sci Eng.

[CR34] Marchant R, Banat IM (2014) Protocols for measuring biosurfactant production in microbial cultures. In: Hydrocarbon and Lipid Microbiology Protocols

[CR35] Mnif I, Ghribi D (2015). Review lipopeptides biosurfactants: mean classes and new insights for industrial, biomedical, and environmental applications. Biopolymers.

[CR36] Mubarak MQE, Hassan AR, Hamid AA, Khalil S, Isa MHM (2015). A simple and effective isocratic HPLC method for fast identification and quantification of surfactin. Sains Malaysiana.

[CR37] Mukherjee AK, Das K (2010). Microbial surfactants and their potential applications: an overview. Adv Exp Med Biol.

[CR38] Mukherjee S, Das P, Sen R (2009). Rapid quantification of a microbial surfactant by a simple turbidometric method. J Microbiol Methods.

[CR39] Mulligan CN (2005). Environmental applications for biosurfactants. Environ Pollut (Barking, Essex 1987).

[CR40] Raaijmakers JM, de Bruijn I, de Kock MJ (2006). Cyclic lipopeptide production by plant-associated *Pseudomonas* spp.: diversity, activity, biosynthesis, and regulation. Mol Plant-Microbe Interact.

[CR41] Rao KY, Lin H, Wu W, Tzeng Y (2008). Evaluation of HPLC and MEKC methods for the analysis of lipopeptide antibiotic iturin a produced by *Bacillus amyloliquefaciens*. Int J Appl Sci Eng.

[CR42] Rao M, Wei W, Ge M, Chen D, Sheng X (2013). A new antibacterial lipopeptide found by UPLC-MS from an actinomycete *Streptomyces* sp. HCCB10043. Nat Prod Res.

[CR43] Romero D, de Vicente A, Rakotoaly RH, Dufour SE, Veening J-W, Arrebola E, Cazorla FM, Kuipers OP, Paquot M, Pérez-García A (2007). The iturin and fengycin families of lipopeptides are key factors in antagonism of *Bacillus subtilis* toward *Podosphaera fusca*. Mol Plant-Microbe Interact.

[CR44] Rudden M, Tsauosi K, Marchant R, Banat IM, Smyth TJ (2015). Development and validation of an ultra-performance liquid chromatography tandem mass spectrometry (UPLC-MS/MS) method for the quantitative determination of rhamnolipid congeners. Appl Microbiol Biotechnol.

[CR45] Satpute SK, Bhawsar BD, Dhakephalkar PK, Chopade BA (2008). Assessment of different screening methods for selecting biosurfactant producing marine bacteria. Indian JHournal Mar Sci.

[CR46] Satpute SK, Banpurkar AG, Dhakephalkar PK, Banat IM, Chopade BA (2010). Methods for investigating biosurfactants and bioemulsifiers: a review. Crit Rev Biotechnol.

[CR47] Smyth TJ, Rudden M, Tsaousi K, Marchant R, Banat IM (2014) Protocols for the isolation and analysis of lipopeptides and bioemulsifiers. In: Springer Protocols Handbooks

[CR48] Smyth TJP, Perfumo A, McClean S, Marchal R, Banat IM (2010) Isolation and analysis of lipopeptides and high molecular weight biosurfactants. In: Handbook of Hydrocarbon and Lipid Microbiology, pp 3687–3704

[CR49] Soberon-Chavez G, Miller-Maier RM (2011) Biosurfactants: A general overview. In: Biosurfactants. From Genes to Applications, pp 1–11

[CR50] Tang J-S, Zhao F, Gao H, Dai Y, Yao Z-H, Hong K, Li J, Ye W-C, Yao X-S (2010). Characterization and online detection of surfactin isomers based on HPLC-MSn analyses and their inhibitory effects on the overproduction of nitric oxide and the release of TNF-α and IL-6 in LPS-induced macrophages. Mar Drugs.

[CR51] US FDA (1999) Guidance for Industry, Validation of Analytical Procedures. Methodology

[CR52] Willenbacher J, Zwick M, Mohr T, Schmid F, Syldatk C, Hausmann R (2014). Evaluation of different *Bacillus strains* in respect of their ability to produce surfactin in a model fermentation process with integrated foam fractionation. Appl Microbiol Biotechnol.

[CR53] Yi G, Liu Q, Lin J, Wang W, Huang H, Li S (2016). Repeated batch fermentation for surfactin production with immobilized *Bacillus subtilis* BS-37: two-stage pH control and foam fractionation. J Chem Technol Biotechnol.

[CR54] Yokota K, Yatsuda M, Miwa E, Higuchi K (2012). Comperative study on sample preparation methods for the HPLC quantification of iturin from culture supernatant of an antagonistic *Basillus* strain. J Int Soc Southeast Asian Agric Sci.

[CR55] Youssef NH, Duncan KE, Nagle DP, Savage KN, Knapp RM, McInerney MJ (2004). Comparison of methods to detect biosurfactant production by diverse microorganisms. J Microbiol Methods.

[CR56] Yuan J, Raza W, Huang Q, Shen Q (2011). Quantification of the antifungal lipopeptide iturin a by high performance liquid chromatography coupled with aqueous two-phase extraction. J Chromatogr B Analyt Technol Biomed Life Sci.

[CR57] Zhu L, Xu Q, Jiang L, Huang H, Li S (2014). Polydiacetylene-based high-throughput screen for surfactin producing strains of *Bacillus subtilis*. PLoS One.

